# Structural control and depth clustering of extensive hydrothermal venting on the shelf of Milos Island

**DOI:** 10.1038/s41598-025-26398-y

**Published:** 2025-11-27

**Authors:** Paraskevi Nomikou, Konstantina Bejelou, Andrea Koschinsky, Christian dos Santos Ferreira, Dimitrios Papanikolaou, Danai Lampridou, Stephanos P. Kilias, Eirini Anagnostou, Marcus Elvert, Clemens Röttgen, Joely M. Maak, Alissa Bach, Wolfgang Bach, Areti Belka, Evgenia Bazhenova, Karsten Haase, Charlotte Kleint, Effrosyni Varotsou, Palash Kumawat, Erika Kurahashi, Jianlin Liao, Eva-Maria Meckel, Ignacio Pedre, Wiebke Lehmann, Enno Schefuß, Michael Seidel, Sotiria Kothri, Solveig I. Bühring

**Affiliations:** 1 Department of Geology and Geoenvironment, National and Kapodistrian University of Athens, Athens, Greece; 2 School of Science, Physics & Earth Sciences, Constructor University Bremen, Bremen, Germany; 3 Faculty of Geosciences, University of Bremen, Klagenfurter Str. 4, 28359 Bremen, Germany; 4 MARUM- Center for Marine Environmental Sciences, University of Bremen, Leobener Str. 8, 28359 Bremen, Germany; 5 GeoZentrum Nordbayern, Friedrich-Alexander-University Erlangen-Nuernberg, Erlangen, Germany; 6 Institute for Chemistry and Biology of the Marine Environment (ICBM), Carl Von Ossietzky University of Oldenburg, Oldenburg, Germany

**Keywords:** Geochemistry, Natural hazards, Solid Earth sciences, Geochemistry, Geomorphology, Tectonics, Volcanology

## Abstract

High-resolution geophysical surveys, complemented by chemical, physical, and visual data collected via the ROV MARUM-SQUID, revealed a previously undocumented intermediate-depth hydrothermal vent field (30–230 m) on the shelf of Milos Island, Greece. Three primary vent areas—Aghia Kiriaki, Paleochori–Thiorychia, and Vani—are aligned with ENE–WSW and NW–SE trending active faults, which define the SE coast of Milos and the Milos Gulf–Fyriplaka graben, deepening the Milos shelf up to 230 m. All areas exhibit a distinct bimodal vent distribution, with peaks at ~ 130 m and ~ 180 m; the Paleochori–Thiorychia region additionally hosts a cluster near 210 m. Shallow vents occur on flat, sandy substrates with white microbial mats and gas emissions, whereas deeper vents feature extensive microbial mats, CO₂-degassing chimneys, and actively boiling fluids exceeding 180 °C, indicative of persistent hydrothermal activity. Although the cause of the bimodal —and, in one case, trimodal—distribution remains uncertain, our findings offer a comprehensive, high-resolution characterization of the spatial distribution, morphology, and fluid characteristics of intermediate-depth hydrothermal venting on the Milos shelf.

**Supplementary Information:**

The online version contains supplementary material available at 10.1038/s41598-025-26398-y.

## Introduction

Research on shallow hydrothermal systems^1^ has traditionally focused on sites within SCUBA-accessible depths (≤ 50 m), leaving medium-depth systems comparatively unexplored. The hydrothermal system off Milos offers a promising opportunity to bridge the knowledge gap between shallow-water and deeper hydrothermal systems^2^. Pronounced geothermal gradients in this area drive vigorous hydrothermal circulation, strongly influenced by magmatic degassing. The emitted CO₂ is partially mantle-sourced and linked to slab-flux processes involving recycled subducted marine carbonates^3,4^. This dynamic system is evident in the hydrothermal fluids of the shallow waters surrounding Milos, with conspicuous white patches composed of sulfur (50–80%) and silica, likely formed through microbial processes^4–6^.

Coastal hydrothermal vents near Milos have been extensively studied over the past decades^4–10^. Some vents emit high-temperature fluids, often accompanied by free gas and aqueous solutions with variable chlorinity and low pH^9,10^. The fluids contain dissolved salts and gases, including CO₂, H₂, H₂S, and CH₄, and undergo varying degrees of seawater mixing. Some fluids are up to 47% enriched in Cl relative to seawater and depleted in Mg²⁺ and SO₄²⁻, and furthermore markedly enriched in arsenic and sulfide^9^. These unique chemical conditions promote the formation of yellow, amorphous arsenic-sulfide precipitates around the vents^11^ and the production of arsenic-sulfur nanoparticles, which constitute a significant portion of the soluble arsenic fraction^11,12^.

While most prior studies have focused on shallow-water venting (e.g., < 100 m depth), evidence for hydrothermal activity at intermediate depths has also been reported. Hydrothermal venting on the Milos shelf has been previously mapped^4,5^, with early surveys describing gas plumes and vent structures at ~ 100 m depth. Subsequent work documented activity between 90 and 350 m in the neotectonic graben south of Milos^13^, while more recent studies have referenced venting extending to nearly 500 m, though direct observations in that case were limited to shallower depths^14^. These studies provide important context, but detailed seafloor mapping and characterization of vent morphology and fluid composition at intermediate depths remain limited.

This study presents the distribution of previously uncharted vents at intermediate depths (30–230 m) around Milos and demonstrates—through the integration of high-resolution bathymetric mapping, seismic reflection data, visual observations, and geochemical measurements—that the Hydrothermal Vent Fields (HVFs) on the Milos continental shelf exhibit a bi- to trimodal distribution. Our results provide a high-resolution characterization of the spatial extent, morphology, and fluid characteristics of these intermediate-depth hydrothermal systems, as well as their association with tectonic structures on the Milos shelf.

## Geological setting

**Fig. 1:**
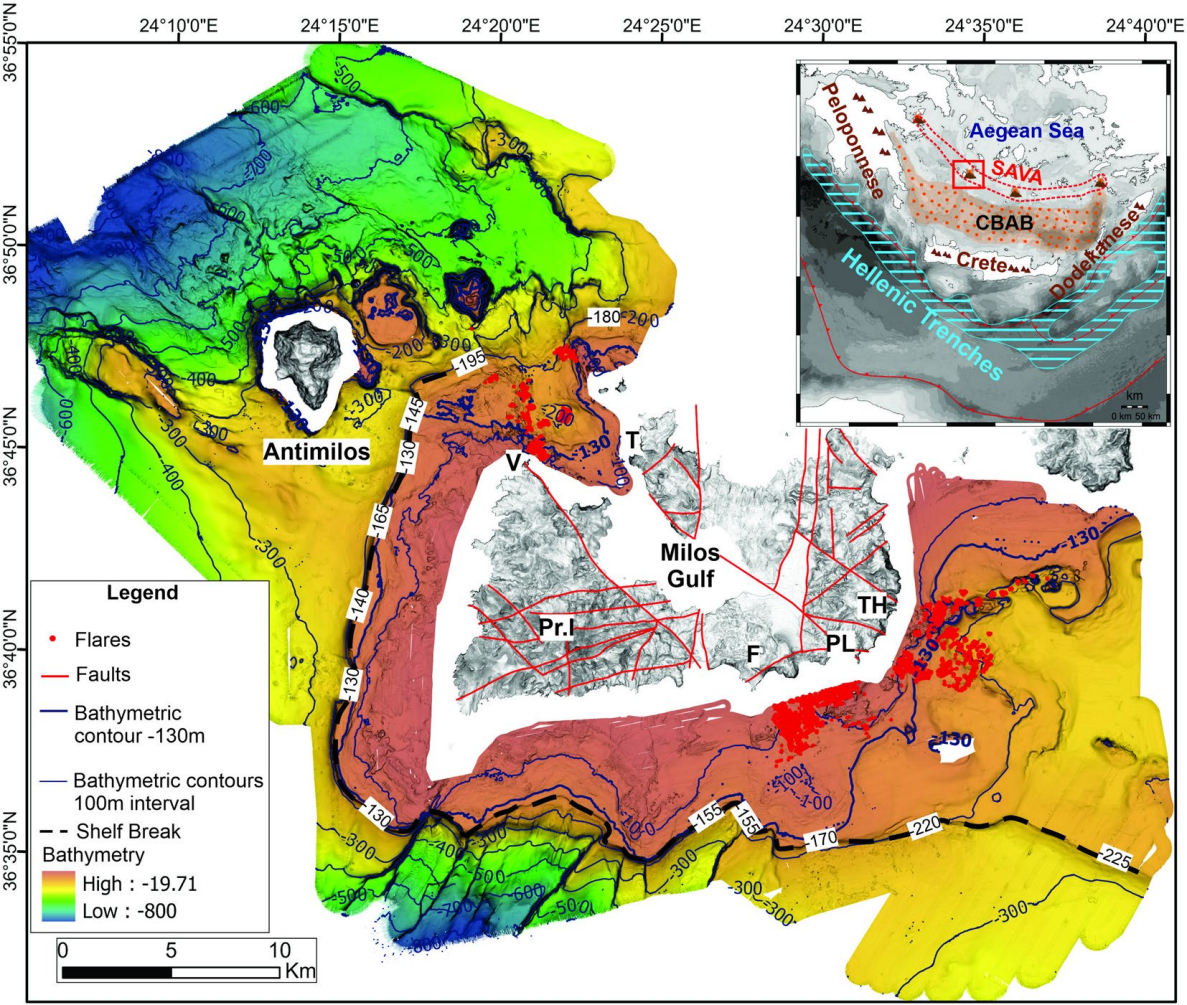
Bathymetric map of the studied area around Milos Island with 20 m contours. The 130 m depth contour is shown in dark blue, and the shelf-break as a thick black dashed line at varying depths. The detected flares interpreted as sites of active gas/hydrothermal venting are shown as red circles. Onshore topography is shown with shaded relief, and the very shallow coastal zone (< 40 m depth) is left white. Abbreviations - V: Vani, Pr. I: Profitis Ilias, F: Fyriplaka, PL: Paleochori, TH: Thiorychia and T: Trachilas. Inset map: The geotectonic frame of the South Aegean Volcanic Arc (SAVA) with the position of the Milos volcanic group (red rectangle) developed at the back-arc area of the Hellenic arc behind the Hellenic trenches, the Peloponnese-Crete-Dodekanese island arc and the Cretan Back-Arc Basin (CBAB)^15^.

Milos is part of the modern South Aegean Volcanic Arc (Fig. 1, inset map), formed due to the African plate subducting beneath the Aegean micro-plate^15^. The oldest (Late Pliocene-Early Pleistocene) volcanic rocks occur in the western parts of Milos and in the neighbouring islands to the east followed by subaerial eruptive products of early-middle Pleistocene age^16–18^. The most recent subaerial eruption occurred 80 thousand years ago in Fyriplaka, leaving a well-preserved crater in the central south region^16,19–21^.

The neotectonic evolution of Milos during Pliocene-Pleistocene involved alternating phases of extension and compression associated with volcanic activity^22^. Detailed neotectonic analysis and mapping of Milos resulted in distinct uplifted or subsided blocks, which are tilted or rotated around inclined axes^23^. The most dominant tectonic features, affecting the overall morphology of the island, are two major fault zones bounding the central block of the Milos – Fyriplaka tectonic graben, hosting the Fyriplaka volcanic crater and numerous vent fields both onshore (Aghia Kiriaki) and offshore near the coast of Paleochori. Seismic events, such as the 5.2 ML on March 20, 1992, in the southeastern part of the graben, had severe impacts, including structural damage and accompanying phenomena (variations in gas emissions, fractures, landslides, etc.)^24^.

Faults of the island have served as conduits for geothermal fluids and have resulted in extensive hydrothermal alteration of the adjacent volcano-sedimentary formations, giving rise to a rich spectrum of exploitable industrial mineral and metal ore-types onland.

## Results

### Acoustic water column data and Geomorphological analysis reveal HVFs around Milos

Acoustic data provided detailed information on the seafloor bathymetry and the occurrence of flares in the water column, indicative of possible hydrothermal venting in three key areas (Fig. 1): Aghia Kiriaki (South offshore Milos, Cluster A), Paleochori-Thiorychia (“Sulfur Mines”, SE offshore Milos, Cluster B) and Vani (NW offshore Milos, Cluster C).

The newly mapped vent fields are exclusively situated on the shelf of Milos Island at depths ranging from the shallow coastal zone to approximately 230 m (Fig. 1, Supplementary Fig. 1). The southwestern portion of the Milos shelf area remains relatively stable, with depths between 130 and 155 m, whereas the areas to the east have subsided to 170–220 m depth (Fig. 1). Active faulting has created the NW-SE Milos Gulf-Fyriplaka tectonic graben where the Aghia Kyriaki (southeast) and the Vani (northwest) vent fields are located. South of the ENE-WSW active faults along the coast of Thiorychia, the SE coast of Milos has subsided^24^.

Hydrothermal activity in the offshore area of the South Aegean Volcanic Arc remains underexplored, particularly beyond the very shallow coastal zones^7^. Notable exceptions include flares within the Santorini caldera at 150–200 m depth^25^ and hydrothermal chimneys with associated flares at the bottom of the Kolumbo volcanic crater at 504 m depth^26,27^. Submersible dives with “Thetis” of the Hellenic Centre for Marine Research have detected several volcanic craters around the Nisyros volcanic field at various depths of 230–450 m depth without hydrothermal activity^28,29^. Thus, the Milos HVFs developed on the shelf down to 230 m depth may be unique features among the submarine volcanic outcrops of the South Aegean Volcanic Arc^30^. A distinction among the flares in the three areas around Milos Island can be made based on their depths relative to the 130-meter isobath (Fig. 1, Supplementary Fig. 1). This distinction may have a significant impact on the geometry and geochemistry of the associated fluids and gases, thereby influencing their biological and mineral characteristics. Ongoing analyses of materials collected during the recent cruise aim to further elucidate these aspects.

**Fig. 2:**
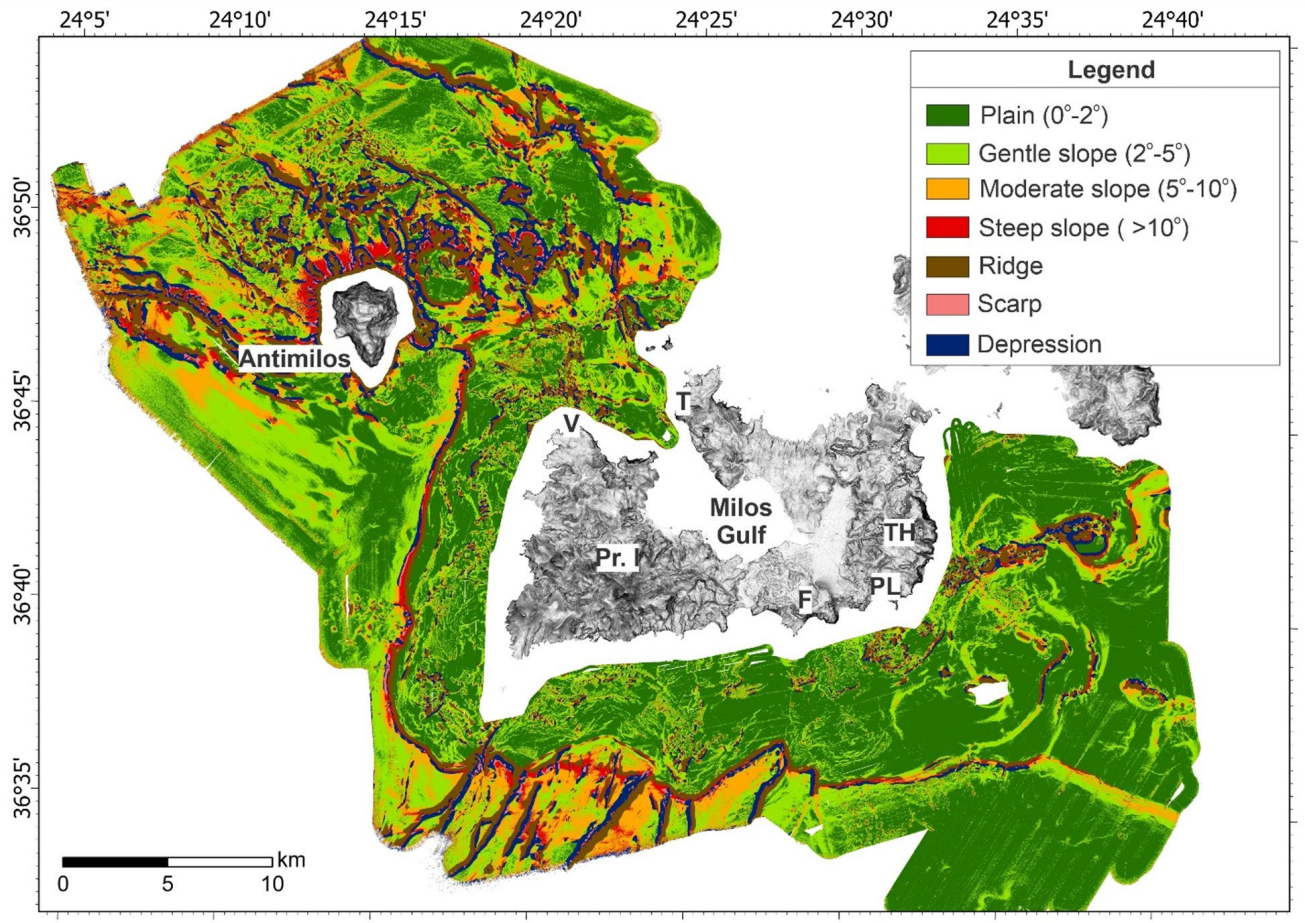
Seabed classification map of the area around Milos. Seven distinct seabed classes have been identified around Milos by the BTM as shown in the legend. Abbreviations - V: Vani, Pr. I: Profitis Ilias, F: Fyriplaka, PL: Paleochori, TH: Thiorychia and T: Trachilas.

Benthic Terrain Modelling Tool (BTM) has been used to categorize seven distinct seabed classes, broadly defining three main areas: (1) the continental shelf and its shelf edge around Milos, (2) the continental slope, and (3) the deeper areas (Fig. 2). The continental shelf surrounding Milos exhibits significant variation in width and complexity, typically characterized by convex or concave topography (ridges and valleys accordingly), high rugosity, and well-developed plains. A notable feature to the east is a distinct linear zone with circular to semi-circular ridges and depressions, trending WSW-ENE, where a significant field of gas flares has been documented^4^.

The seaward extension of the shelf, referred to as the shelf edge, is distinctly classified as a ridge, with depths ranging from 80 m to 235 m, and is often bounded by steep slopes. The southern portion of the shelf edge is of particular interest, as it deepens in alignment with an onshore fault striking NW-SE through Milos. Similarly, the northern part of the shelf edge is also found at greater depths between the two faults running NW-SE across the island (Fig. 1).

Linear ridges, depressions, and high slope values characterize the southwest region of the mapped area. The morphology and presence of carved embayments along the shelf are interpreted as the result of tectonically controlled landslides. Northwest of Milos, the seabed morphology, especially around Antimilos, is highly complex due to volcanic activity^13^. Several circular ridges have been identified in this area (Fig. 2), which were previously described as volcanic domes^13^ in the case of larger formations, and hummocks in the case of smaller ones.

### Distribution and characteristics of the newly mapped HVFs

The HVFs were mapped using the multibeam echosounder systems of the R/V *Meteor* and of the autonomous underwater vehicle (AUV) MARUM-SEAL and were visually documented and sampled using the remotely operated vehicle (ROV) MARUM-SQUID (Supplementary video).

**Fig. 3:**
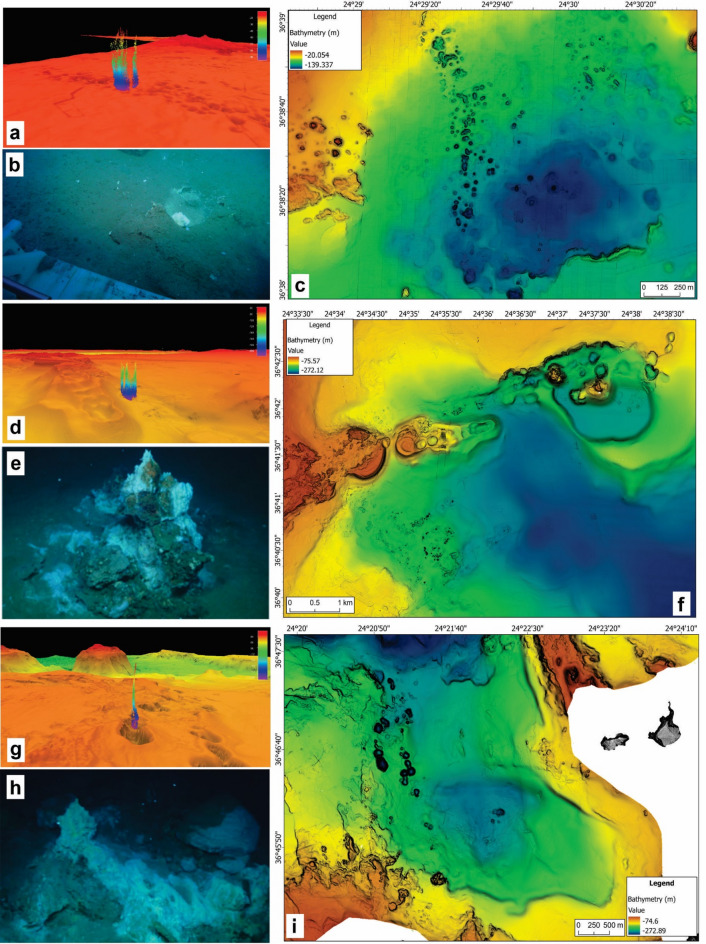
Ag. Kiriaki (**A**): a) water column depiction above the illustrated area, coordinates: 36°38.824’ N, 24°29.4516’ E, b) ROV photo above the seafloor at the area of Ag. Kiriaki with patches of bacterial mats on the sediments, c) AUV bathymetric map illustrating the morphology of the depressions formed due to hydrothermal activity. Paleochori-Thiorychia (**B**): d) water column depiction above the “Celestial Chimney”, coordinates: 36°42.0233’ N, 24°35.6548’ E, e) ROV photo of the “Celestial Chimney” partly covered with white bacterial mat (GeoB255-20), f) AUV bathymetry combined with multibeam data to illustrate the morphology of the seafloor east of Paleochori-Thiorychia. Vani (**C**): g) water column depiction above the illustrated active area of Vani, h) ROV photo above a highly active area with boulders covered partly with bacterial mat and yellow sulfur precipitated (GeoB255-36), coordinates: 36°46.6873’ N, 24°20.9174’ E, i) AUV bathymetric map illustrating the morphology of the vent field within the Vani graben.

*Aghia Kiriaki (A)*: The submarine area south of Ag. Kiriaki consists of a flare field (Fig. 3a) along a NW-SE zone, starting at ~ 90–100 m depth (Fig. 3c). The southwestern edge of this field (to 115 m depth) is oriented in a N-S direction that follows the western border of a wider depression (E-W diameter: ~1700 m, N-S diameter: ~1000 m). This N-S oriented portion, approximately 2000 m long and 300 m wide, contains evenly distributed small depressions (every 10–30 m) with walls ranging from 4 to 8 m deep (Supplementary Fig. 2).

The Aghia Kyriaki hydrothermal field features fine-grained sediment with localized diffuse venting, often covered with white, flocculent microbial mats^31^ (GeoB255-17; Fig. 3b), including occasional occurrence of red precipitates and terraces of indurated sediment (Supplementary video). Gas emissions range from fine streams of bubbles to vigorous degassing, creating small eruptive holes. Temperatures measured 20 cm into the sediment below small mounds of indurated whitish sediments that were releasing gases reached up to 132 °C.

In the southern part of the field at ~ 100 m depth, flat areas with fine-grained, brownish-grey sediments exhibit terraces of indurated sediment with wide white spots and gas emissions (GeoB255-26). A presumably old hydrothermal site was identified by a porous chimney structure, now biologically encrusted on an otherwise flat sandy sediment.

To the north, at 107 m depth, a flat area exhibits white and red surface patches. Further northeast, at 105 m depth, a ~ 1 m high hydrothermal chimney with shimmering fluids, white mats and red minerals was located. Shimmering fluids of up to 72 °C were also sampled from indurated sediment layers, with white mats and gas emissions. The temperature of fluids in this area were measured up to 72 °C, with pH values around 6, while reaching redox potential (Eh) values as low as −190, and hydrogen concentrations up to 300 nmol l⁻¹ (Table 1).

**Fig. 4:**
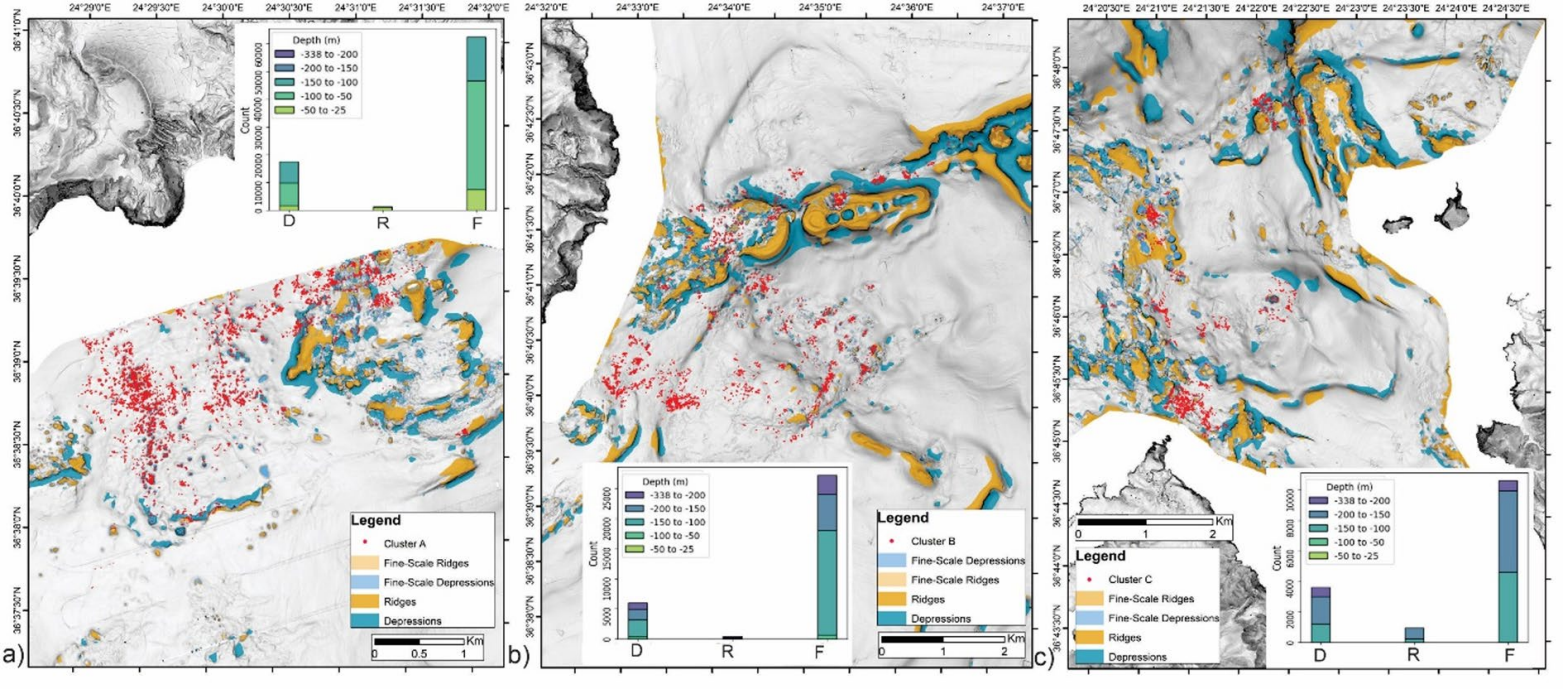
Seabed Classification map of Ag. Kyriaki (**a**), Paleochori-Thiorychia (**b**) and Vani (**c**) herein referred as Cluster A, B and C respectively including inset histograms depicting the correlation between gas flares and seabed classification (D: Depression, R:Ridge, F:Flat area). Correlation between gas expulsion and seabed geomorphology was conducted by associating gas release locations with specific morphological features identified through the seabed classification scheme, namely depressions, ridges, and flat areas. The analysis reveals that the majority of gas flares occur in flat areas, followed by depressions.

Correlating seabed classification with gas flares distribution revealed that the majority of gas flares occur in flat areas, followed by depressions (Fig. 4a). A smaller portion of flares were observed along ridges, which are associated with constructional features. Specifically, in Aghia Kyriaki, the ridges correspond to the shallow, rugged terrain (Fig. 4a).

#### Paleochori-Thiorychia (B)

This area features a distinct flare field (Fig. 3d) along an ENE-WSW trending, sword-shaped ridge, with base depths ranging from 150 m to 230 m (Fig. 3f). It is more than 4.5 km long and it is characterized by the existence of dome- and crater-shaped features in a terraced configuration. Starting from the west, the largest feature is a circular dome with a diameter of ~ 1000 m. An elongated crater forms the tip of the ridge; its diameter along its long axis (NE-SW) is ~ 600 m and along the short one (NW-SE) is ~ 215 m.

During the ROV survey the most notable features mapped were the “Celestial Chimney” at 206 m depth (GeoB255-20; Fig. 3e), emitting fluids at 56 °C, and “Little Chimney Garden of Thiorychia,” comprising ~ 10 slender chimneys, up to 50 cm tall, with fluid temperatures of up to 25 °C (Supplementary video). The relatively low temperatures at these sites were accompanied by pH and Eh values closer to ambient seawater (Table 1), suggesting less chemically altered fluids compared to other deeper vent sites. While we cannot fully exclude technical limitations or sampling issues as contributing factors, similar patterns have been observed in shallow coastal vents near Milos, where lower temperatures and near-seawater fluid signatures were attributed to increased seawater incursion into the hydrothermal circulation system^14^. These findings emphasize the heterogeneity of vent characteristics, even at similar depths or within the same fault-controlled system, and highlight how local permeability, fluid flow paths, and seawater mixing can strongly influence vent expression across short spatial scales.

A protruding overhang, named “White Sealhound” (because of its resemblance to a floppy-eared dog with the body of a seal), measuring 1 m high and 2.2 m length, see orthomosaic and digital elevation model (supplementary material: 10.1594/PANGAEA.974816), was entirely covered with white mats (GeoB255-21/24; Supplementary video). Fluid temperatures here reached 180 °C, pH was at 5.6, Eh at −180 and the hydrogen concentrations were extremely elevated (3857 nmol l^− 1^, Table 1)^32^. Adjacent areas featured indurated vertical faces with horizontal bedding and various white mat types at sites of gas emissions.

The correlation between gas expulsion and seabed geomorphology revealed that, unlike in Ag. Kyriaki (A), gas flares in Paleochori–Thiorychia (B) were detected not only in shallow, rugged areas but also on isolated, morphologically elevated features and predominantly in flat regions (81% compared to 76% in Ag. Kyriaki) (Fig. 4b).

**Fig. 5:**
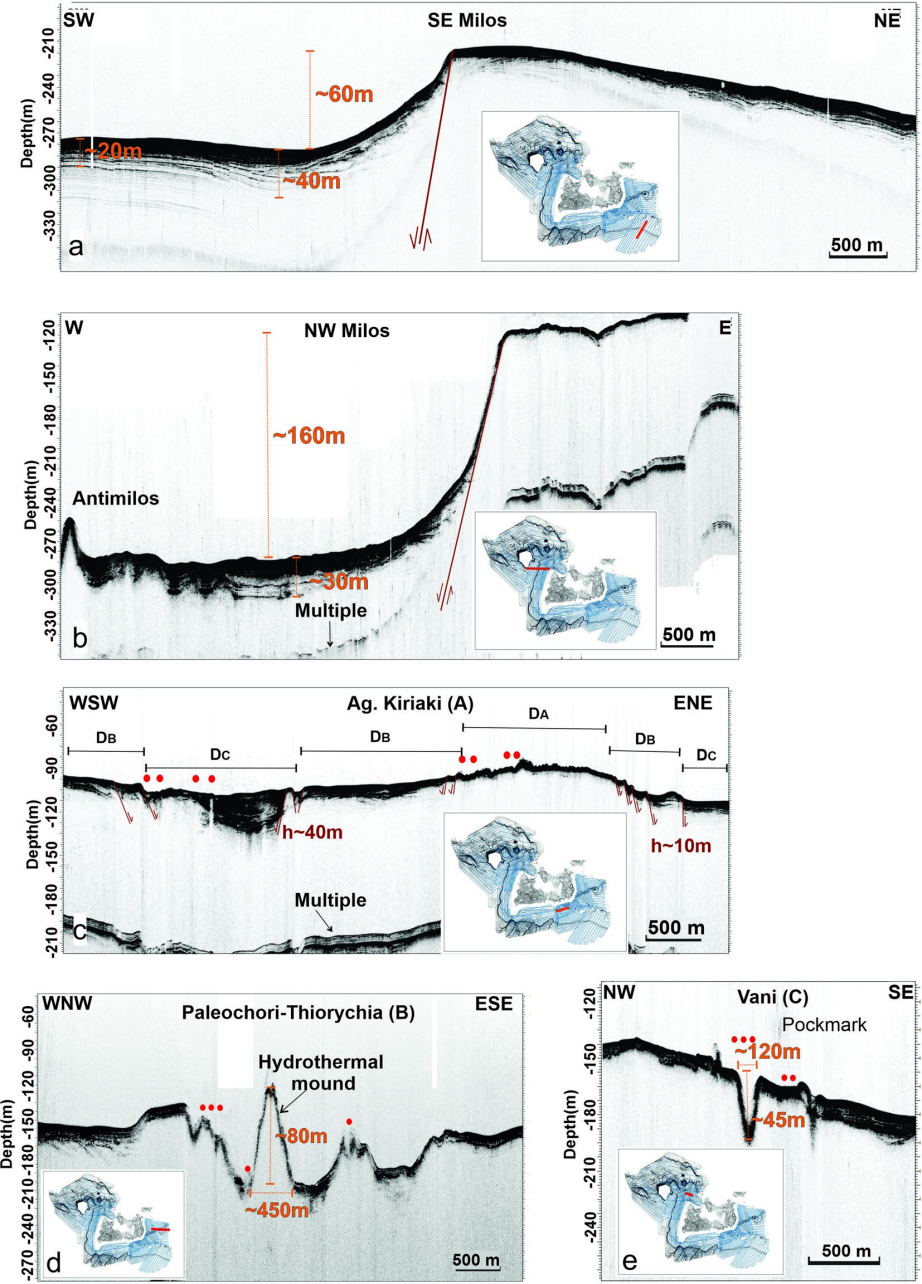
Seismic line across the shelf break of (**a**) the SE area off Milos, (**b**) NW area off Milos, (**c**) the Aghia Kiriaki (A) vent field south off Milos, including indications of the detected flares (red spots). Seismic line across the (**d**) Paleochori-Thiorychia (B) vent field SE off Milos with indication of a large mound (80 m high) and the (**e**) Vani (C) vent field NW off Milos with indication of a large pock mark (45 m deep).

*Vani (*C*)*: Approximately 35 km north of Cape Vani on Milos, a cluster of gas flares (Fig. 3g) aligned along a NW-SE trending zone, approximately 700 m wide marks the western border of a deeper basin extending southeastwards towards Milos Bay (Fig. 3i). This area features a series of depressions, the western of which are relatively uniform, with diameters ranging from 185 to 200 m, while the eastern ones vary from a few tens of meters up to 150 m (Supplementary Fig. 2). The shallowest rim of the western depressions range between 170 and 190 m water depth and their floors at depths of 180–230 m. The eastern group is deeper, with rim at 180–200 m and floors at 190–220 m (Fig. 5e).

ROV dives along a fault zone revealed tectonic activity and significant gas emissions. The shallower part of the Vani hydrothermal field has a flat sandy seabed partly covered with white mats, and brown precipitates (GeoB255-34). The escaping fluids had temperatures of around 18 °C with pH around 7.5 and very low Eh values of below − 200 (Table 1). Numerous gas seeps were identified, including continuous vigorous bubble venting, that created visible holes in the seabed.

The deeper, northern sections of the Vani vent field were found on a sandy field with subtle topographic elevations, like craters. Hydrothermal activity was evident in vigorous degassing at a depth of 186 m, where fluid reached temperatures around 100 °C, Eh values of below − 230 and hydrogen concentrations of up to 3549 nmol l^− 1^ (Table 1). Massive, indurated sediment blocks were discovered, resembling “giant Lego-like” structures (Fig. 3h) with abundant hydrothermal activity (GeoB255-36; Supplementary video).

Within the Vani vent field, the majority of gas flares were detected in depressions (24%), exceeding those observed in Ag. Kyriaki (A) (21%) and Paleochori-Thiorychia (B) (18%) (Fig. 4c).

**Table 1:** Characteristics (depth, temperature, pH, Eh and hydrogen concentration of the fluids) of different hydrothermal sites in the areas Aghia Kiriaki, Palaeochori-Thiorichia and Vani.

Area	Depth (m)	T (°C)	pH	Eh	H_2_ (nmol/L)	Coordinates	Description	notes	GeoB#
Aghia Kiriaki	102	21	6	−190	300	36°38,880’N 24°29.446’E	white spots	fluids collected through funnel	25526-9
	106	72	n/a	n/a	54	36°40.1470’N 24°32.8994’E	indurated sediment, shimmering fluids		25526-13/14
	106	26	n/a	n/a	188	36°40.1478’N 24°32.9022’E	indurated sediment, shimmering fluids, vigorous degassing		GeoB25526-9
Paleochori - Thiorychia	203	22	8.15	158	2	36°42.0216’N 24°35.6830’E	white spots	fluids collected through funnel, KIPS problems	25520-2
	206	56	8.21	151	9	36°42.0233’N 24°35.6548’E	*Celestial Chimney*	KIPS problems	25520-2/7/8
	206	25	8.06	114	17	36°42.0265’N 24°35.6588’E	*Thiorychia’s little chimney garden*	KIPS problems	25522-2
	188	68	n/a	n/a	6	36°41.9872’N 24°35.5168’E	*Shimmering boulder pile*	KIPS problems	25522-6/7
	189	180	5.57	−180	3857	36°42.0265’N 24°35.6588’E	*White Sealhound*		*25524-5/6/7*
Vani	129	18	7.5	−205	2	36°45.3464’N 24°21.5717’E	white and brown patches, vigorous degassing	fluids collected through funnel	25534-3
	128	17	7.5	−202	14	36°45.332’N 24°21.573’E	small white mound, string of pearls degassing		25534-7/8
	187	100	6	−232	46	36°46.6873’N 24°20.9174’E	slope with shimmering fluids, vigorous degassing, white mats and yellow precipitates		25536-5
	186	49	6.5	−239	3549	36°46.8600’N 24°20.9808’E	giant lego-like blocks and “construction foam” microbial mats		25536-10/11

Due to morphological, vent style, and mineralogical similarities, these HVFs might be considered as shallower water, and lower temperature, analogues of the CO_2_-degassing, seafloor massive sulfide vents of the deep-sea Kolumbo volcano (500 m depth)^33^.

## Discussion

The distribution of the newly mapped vent fields around Milos Island reveals three areas Ag. Kiriaki (Α), Paleochori-Thiorychia (Β) and Vani (C) covering vast parts of the Milos shelf. The shelf break (Fig. 5a, b) can be traced around the island, with its depth varying according to geographic orientation. Along the western and southwestern sectors of the Milos shelf, the shelf break occurs at approximately 130–150 m, near the 130 m isobath, which corresponds to the typical depth in stable areas unaffected by active faulting. In contrast, the shelf break is disrupted along the eastern outcrops (Paleochori-Thiorychia B), where subsidence has lowered it to depths of approximately 170–220 m.

**Fig. 6:**
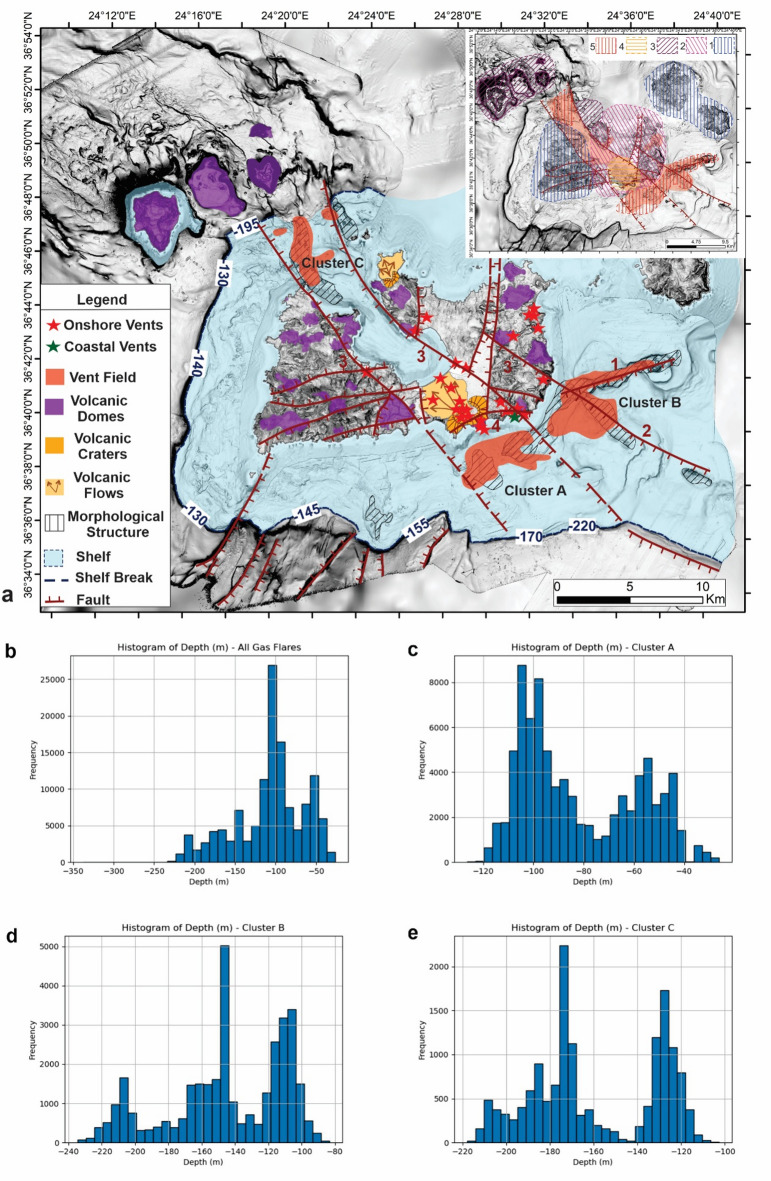
(**a**) Synthetic volcano-tectonic map of Milos and surrounding areas illustrating the prolongation of the main onshore active faults within the surrounding shelf and their relation to the newly discovered vent fields. The map helps to detect also the other neotectonic faults which do not affect the shelf and are not related to the vent fields. It also shows the relation of the last volcanic eruption of Fyriplaka with the NW-SE tectonic graben and the ENE-WSW fault zone of SE Milos with the associated vent fields. The main outcrops of the Plio-quaternary volcanic domes are shown and their relation to the neotectonic and active faulting is indicated by their tectonic disruption. The most active onshore and shallow coastal vent fields are also shown. Faults: 1: ENE-WSW fault, 2: NW-SE Thiorychia fault, 3: NW-SE fault zones, 4: ENE-WSW Fyriplaka volcano fault. Inset map: The five volcanic areas/periods of Milos. 1: Pliocene volcanism (3.5-2.3.5.3 Ma), 2: Early Pleistocene volcanism (1.9–0.9 Ma), 3: Middle Pleistocene volcanism (0.45 − 0.32 Ma), 4: Late Pleistocene volcanism (0.12 − 0.08 Ma), 5: Present hydrothermal activity. (**b**) Depth histogram for all the flares. (**c**) Depth histogram of flares for Cluster A (Aghia Kiriaki). (**d**) Depth histogram of flares for Cluster B (Paleochori – Thiorychia). (**e**) Depth histogram of flares for Cluster C (Vani).

Contrasting morphological hydrothermal features, such as those observed at Paleochori–Thiorychia (B) (large mound) and Vani (C) (large pockmark) (Fig. 5d and e), may result from differences in depositional and dissolution processes, as well as from the distinct geochemical and biological characteristics of the vents. Sedimentation also appears to play an important role, as illustrated by the three domains identified at Aghia Kiriaki (A), which display varying sediment thicknesses—ranging from minimal deposition on crustal material to medium-thickness soft sediments and to maximum-thickness stratified sediments (depositional areas D_A_, D_B_, and D_C_, respectively; Fig. 5c). Flares along this profile occur in areas with thinner sediment cover. Tectonic activity further influences local sedimentation rates, as shown in Fig. 5d and e: sediment thickness in the hanging wall reaches up to ~ 40 m, whereas on the footwall of the shelf it is only a few meters thick (Fig. 5a and b). The detailed mapping of the shelf break shows a topographic relief with drops varying between approximately 50 m and 160 m (Fig. 5a and b). Its morphology is strongly affected by tectonism. On the western block of Milos, the shelf break lies at 130–150 m, typical of pre-Holocene sea-level lowstands. In contrast, within the NW–SE graben in the southeastern sector, it deepens to 170–220 m due to active faulting. This deformation aligns with the continuation of the major NW–SE fault system that bounds the Milos Gulf–Fyriplaka graben both to the southeast and northwest (Fig. 6). Accordingly, Aghia Kiriaki named as Cluster A and Vani named as Cluster C vent fields are bounded by this graben, while the Paleochori–Thiorychia vent field named as Cluster B lies along the eastern extension of the ENE–WSW fault zone of SE Milos, with additional control from the NW–SE Thiorychia fault^24^.

The spatial distribution of gas flares in Cluster A (Supplementary Fig. 3) shows a distinct alignment with the coastline, predominantly following a SW–NE orientation with a secondary N–S trend. This orientation reflects the influence of SW–NE trending faults. The field is situated along the eastern edge of the Fyriplaka Volcano, where NW–SE and ENE–WSW fault zones intersect (Fig. 6), a setting that coincides with one of the most active onshore vent areas on Milos^24,25^.

Cluster B is characterized by two principal orientations (Supplementary Fig. 3): a dominant NNW–SSE trend and a secondary NW–SE trend. The latter is closely associated with a linear morphological feature extending offshore, marked by alternating crater-like depressions and vents. This configuration corresponds well with the onshore fault system, particularly the three major NW–SE fault zones that traverse Milos and are linked to tectonic horsts and grabens formed under a NW–SE extensional stress regime. The corresponding onshore Cluster B vent field is primarily controlled by the ENE–WSW fault zone bounding the south of the Fyriplaka Volcano, with a secondary influence from the NW–SE trending Thiorychia fault (Fig. 6). These structures are consistent with the observed alignment of venting activity offshore^24,25^.

Cluster C is more complex (Supplementary Fig. 3), with a dominant SW–NE trend and a secondary W–E orientation. The vent field lies within the NW–SE tectonic graben of the Milos Gulf–Fyriplaka system, which continues offshore and exerts strong control on vent distribution. The W–E trend observed here correlates with the WSW–ENE trending faults documented in the western part of Milos (Fig. 6).

Overall, comparison of spatial distribution of gas flares with onshore fault structures highlights a strong correlation between gas flare orientations and the regional tectonic fabric (Supplementary Fig. 4). All clusters exhibit influence from SW–NE trending faults, while Cluster B additionally reflects the role of the major NW–SE fault systems, and Cluster C shows evidence of control by W–E to WSW–ENE oriented faults. It is noteworthy that the most active onshore venting is observed at Aghia Kiriaki, Paleochori, and Thiorychia, where fault intersections and major structural trends converge, reinforcing the strong structural control on vent distribution in both onshore and offshore environments.

Analysis of the vent field distribution in relation to active faults and the overall tectonic block structure indicates the possibility of two extensive areas on either side of the Milos Gulf–Fyriplaka tectonic graben that lack geothermal activity, except in the southeastern part of Milos south of the Thiorychia fault (Fig. 6). Notably, the major E–W onshore faults forming the Prophitis Ilias tectonic horst in southwest Milos—where volcanism dates to the Late Pliocene–Early Pleistocene—do not influence the shelf, and no active hydrothermal activity is observed there. Conversely, in the southwestern slope zone at depths of 400–550 m, certain NE–SW faults with throws of 20–60 m (inferred from bathymetric differences across the faults) terminate at the shelf break and do not extend northeast onto the shelf, where hydrothermal activity is absent. Additional geophysical and geological studies are needed to establish whether these structurally distinct zones genuinely lack active fluid flow or were not resolved with the current survey coverage.

The depths of the detected flares have been statistically analyzed, and the histograms illustrate both the overall distribution and the distribution in each area (Fig. 6b). The flares of Cluster A are confined at shallow depths up to 130 m with two distinct peaks at 105 m and 55 m (Fig. 6c). In Cluster B, flare activity extends down to 230 m, with three maxima at 145 m, 110 m and 210 m (Fig. 6d). Similarly, in Cluster C, flares occur at depths down to 230 m, with two peaks at 175 m and 130 m (Fig. 6e). However, the overall depth histogram indicates that the most frequent flare depth is 110 m (Fig. 6b) and only a small percentage lies at depths between 150 and 230 m.

The geometry and structural setting of the newly mapped vent fields reveal two spatially distinct domains on the Milos shelf. The southeastern domain (Aghia Kiriaki (A) and Paleochori–Thiorychia (B)), partially documented in earlier studies, is associated with an ENE–WSW-elongated dome (~ 15 × 5 km). In contrast, the northwestern domain (Vani (C)), previously unrecognized, occupies a ~ 6 km-wide dome-like structure within the Vani tectonic graben—the northwestern segment of the Milos–Fyriplaka graben. These two vent domains are separated by approximately 20 km and occur within distinct tectonic subregions of the Milos volcanic field. While their separation and structural differences may reflect complex subsurface processes, our data do not permit further interpretation of magmatic plumbing or fluid sources. Instead, the mapped vent distribution emphasizes the influence of fault architecture and regional tectonics in shaping hydrothermal activity across the shelf, consistent with the Late Pliocene to present-day volcanic and tectonic history of the area (inset, Fig. 6).

The hydrothermal field we describe on the shelf of Milos adds a valuable new example to the relatively small group of known intermediate-depth vent systems (50–500 m), which occupy a transition zone between shallow coastal and deep-sea hydrothermal environments. A notable analog is Kick’em Jenny (Grenada), an active submarine volcano where hydrothermal vents and CO₂-rich discharge occur at ~ 260–265 m in the crater depression—forming mound-like vents and diffuse flow with temperatures up to ~ 180 °C and intense iron-oxide microbial mats^34^. Alongside this, the El Hierro/Tagoro system (90–215 m) exhibits two depth clusters of active venting and magmatic cones^35^. Other intermediate-depth sites include Kolumbo (~ 500 m)^27^ near Santorini and Iceland’s Strytan field (16–70 m)^36^, the latter fueled by alkaline, groundwater-fed fluids.

Although Milos and Kick’em Jenny both feature CO₂-dominated venting and intermediate depths, their genetic mechanisms diverge significantly. Kick’em Jenny is a volcanic edifice powered by magmatic heat and occurs within a crater-hosted depression, producing mound-like vents and diffuse saline flows. By contrast, the Milos vents lie along tectonically controlled fault zones in a sedimented shelf environment, with chimneys constructed from hydrothermal precipitates—not volcanic ejections. We find no hornito-like structures or lava spatter morphology analogous to Kick’em Jenny or Tagoro. Instead, Milos chimneys appear to form through focused hydrothermal fluid upflow and mineral deposition influenced by basement permeability. While the Milos field shares with Tagoro the presence of CO₂-rich discharge and a bimodal depth distribution of venting, the underlying geological processes and vent morphologies differ significantly. These comparisons highlight the diversity of geological settings that can give rise to hydrothermal activity at intermediate depths. The evolution of the Milos hydrothermal vent fields is marked by their occurrence on a continental shelf that has subsided to depths of up to 230 m as a result of active faulting. The vent distribution exhibits a clear bi- to trimodal pattern closely associated with fault-controlled fluid flow, indicating that tectonic structures exert a primary control on the location and characteristics of hydrothermal activity on the Milos shelf.

## Methods

Systematic mapping using the multibeam echosounder systems of RV Meteor and AUV (Autonomous Underwater Vehicle) MARUM-SEAL in in an area ranging from 10 to 20 km from the coast of Milos Island was performed during cruise M192 of R/V Meteor, 08 August – 05 September 2023.

### Multibeam data

The hull-mounted Kongsberg multibeam echosounder EM710 uses a frequency range of 70–100 kHz, suitable for depths ranging from 3 m below the transducers to approximately 1000 m.

During the M192/1 mission, the EM710 was employed to collect data within four survey areas. The EM710 operated with a frequency range of 70–100 kHz, featuring a footprint of 1°/1°. The maximum ping rate was set up to 40 Hz, and the beam spacing mode to High Density Equidistant, resulting in 800 soundings per ping in dual swath mode.

The Seafloor Information System (SIS) software was used for the acquisition, and the four sound speed profiles were integrated into it. They were acquired during data collection and incorporated into the acquisition software using the CTD onboard of the vessel (Fig. 5.1.1.1 in^31^). Continuous acquisition of bathymetric data took place except for the time when AUV deployment, operation, and recovery missions were underway.

Moreover, along with the bathymetric data (*.all), water column data (*.wci) were gathered to detect acoustic water column backscatter anomalies that might signify potential gas seepage originating from the seafloor.

The bathymetric data processing was performed utilizing Qimera QPS. The processing sequence implemented a spline filter to eliminate inaccurate beams, followed by manual editing. Subsequently, a CUBE surface was generated and then exported in ASCII format.

### Automated underwater vehicle MARUM-SEAL

The MARUM SEAL is an autonomous underwater vehicle (AUV) designed by International Submarine Engineering for deep-sea exploration. It is 5.75 m long, weighs 1.35 tons, and operates using modular components. The AUV - as a dedicated autonomous vehicle ‐ has to be programmed as a mission of targets before being operated under water by using the MIMOSA (©Ifremer) mission‐planning tool as a software package designed to operate underwater vehicles. The AUV SEAL is equipped with advanced sensors, Kongsberg EM2040 multibeam sonar, and a navigation system, including a very precise iXSea PHINS inertial navigation unit - in combination with an RDI 300 kHz Doppler log. iXSea POSIDONIA Ultra-Short Base-Line System and a MARUM developed QGIS PosiView plugin used for supervision, in order to monitor the vehicle’s position at sea surface and underwater. It is furthermore used to implement navigation corrections during missions. During the M192-1 cruise 4 dives were completed. The AUV SEAL 5000 successfully collected water-column-, backscatter- and micro-bathymetry- data, as well as georeferenced CTD sensor data. The detailed maps (resolution 5 m) provided by the shipborne multibeam echosounder EM710 allowed for low flying altitudes, resulting in high resolution (1 m) AUV data. In total, tracklines covered 198 km distance, which mapped 17 km^2^ seafloor (Fig. 5.4.1.1–3.1 in^31^). Most of the surveys were conducted with a line spacing of under 100 m at an altitude of 40 m above the seafloor using 400 kHz frequency. Water column data (WCD) were recorded in order to detect possible gas flares.

Raw data collected by the AUV SEAL 5000 included .all (bathymetry) and .wcd (water column) files. Post-processing of the raw bathymetry and navigation data was performed using the MB-System software v.5.7.9beta52, resulting in 1-m horizontal resolution bathymetric maps. Water-column data were replayed using the QPS FMMidwater software, to identify the geographic (Lat, Lon) locations of potential gas/hydrothermal flares. Beam and along-track stack views were utilised. This was of particular importance for the ROV mission planning performed during M192/2 (Leg 2).

### Data Preparation

The two bathymetric grids obtained from the Multibeam Echosounder (MBES) and the Autonomous Underwater Vehicle (AUV), with spatial resolutions of 5 m and 1 m respectively, have been integrated into a single unified Digital Terrain Model (DTM). A resampling step was necessary to align the datasets onto a common grid. In this process, the 5 m raster was resampled to match the 1 m resolution, resulting in a combined raster at 1 m grid spacing. It should be noted that, although the nominal resolution of the resampled raster is 1 m, this finer cell size does not add spatial detail to the original 5 m dataset. The resampling merely interpolates the original 5 m values onto a 1 m grid, so the underlying information content of the coarser dataset remains unchanged.

### Seabed classification

Two distinct seabed classification schemes were employed, each addressing different spatial scales. The first classification was conducted across the entire mapped area, focusing on large geomorphological structures such as ridges, flat areas, and regions with moderate slope values. The second, more detailed classification was applied specifically to areas where gas flares were detected. This finer-scale classification aimed to delineate small-scale morphological features that could be correlated with gas release and subjected to further statistical analysis. All classification processes were carried out using ArcMap 10.8 and ArcPro 3.3, utilizing the Benthic Terrain Modeler tool and the Ga_SaMMT tool.

### Fry analysis

The Fry analysis was applied to the mapped gas flare locations in order to investigate their spatial distribution and determine whether they exhibit preferred orientations or alignments. This statistical method helps reveal whether the points (gas flares) show a random pattern or if they are systematically correlated with tectonic lineaments in the study area. Each gas flare was then taken as a reference, and the relative positions of all other flares were re-plotted around it to generate Fry diagrams. These diagrams were superimposed to produce a composite plot, from which elliptical or linear patterns were identified and compared with the orientations of regional tectonic lineaments.

### Visualization and statistical analysis

Statistical analyses of depression geometry, depth, and gas flare distribution were performed using Python 3.7 to quantify spatial and depth-related patterns. Seabed features were classified into depressions, ridges, and flat areas, and depth values were grouped into defined bathymetric intervals. Stacked histograms were generated to visualize the frequency of these features with depth, while additional plots of gas flares versus depth were produced to assess their vertical distribution and potential correlation with seabed morphology.

### Parasound data

The deep-sea parametric sub-bottom profiler (SBP) known as the hull-mounted ATLAS PARASOUND P70 operates on the principle of the parametric effect, leveraging the non-linear relationship between pressure and density during sonar transmission. To achieve this, it employs two powerful waves with frequencies approximately ranging from 18 to 20 kHz (referred to as primary high frequency, PHF) and an additional 22–24 kHz wave. These combine to produce a secondary high frequency (SHF) (around 40–42 kHz) and a secondary low frequency (SLF) of about 4 kHz. The SLF is utilized for sub-bottom profiling, while the PHF signal can be simultaneously recorded to visualize potential gas bubbles, plankton, fish, or nepheloid layers within the water column. The transducer array has an opening angle of 4° by 5°, resulting in a footprint size equivalent to about 7% of the water depth (Supplementary Fig. 5).

The software ATLAS PARASTORE is used for storing and displaying echograms. Several file formats are recorded during PARASOUND operations: Original *.asd files, which were replayed in PARASOUND for further processing, contain data of the entire water column as well as the sub seafloor. PARASTORE also produces *.ps3 files recorded along with the auxiliary data and *.segy files. All data were loaded into the seismic interpretation software IHS Kingdom and transformed from amplitude to envelope data for further interpretation.

### Remote operated vehicle (ROV) MARUM-SQUID

MARUM-Squid is a 2000 m depth rated, light work-class ROV manufactured by SAAB-Seaeye (UK). The entire system (ROV, winch, power supply and topside control) is shipped inside a single 20” ISO container. The *Leopard* type ROV system was adapted and modified for marine research by MARUM and put into service in January 2016. For the visual documentation of organisms or processes on the seafloor, MARUM-Squid is equipped with a 4 K color zoom camera. The specialized lens corrects chromatic, geometric and radial distortion (4 K corner-to-corner resolution) when using optical systems underwater. An advanced navigation sensor package allows the positioning and displacement of the vehicle with an accuracy of 10 cm along the seafloor. The 7-function, fully proportional manipulator can be used to operate a variety of scientific tools or to collect samples from the seafloor. For the Meteor Cruise M192/2, fluid^37^ and gas tight samplers^38^ were installed on the vehicle, together with other tools such as pushcorer, T-logger and 5 L Niskin bottles.

### pH and Eh

Onboard measurements of pH (WTW^®^ pH-Electrode SenTix^®^ 940), and Eh (WTW^®^ Electrode SenTix^®^ORP-T 900) were conducted at room temperature of 21–22 °C immediately after recovery of the samples.

### Hydrogen

The concentrations of dissolved hydrogen (H₂) were quantified using a reducing compound photometer (RCP; Peak Performer 1, Peak Laboratories, USA). For each measurement, 40 mL of water sample was drawn into a syringe, then 10 mL of high-purity nitrogen (N₂) was added to create a headspace. The syringe was shaken vigorously for 60 s to equilibrate the dissolved gases with the headspace. An aliquot of the headspace gas was immediately withdrawn with a second syringe and injected into the RCP at least twice. To verify the performance of the device and calibrate the system, H₂ gas standards with different concentrations were analyzed every day before operation.

### Software

Figures 1, 2 and 4, and 6 were produced using ArcGIS Pro 3.5 (https://www.esri.com/en-us/arcgis/products/arcgis-pro/overview). The inset maps in Fig. 4 were generated with Python 3.4. Figure 3 presents bathymetric data and ROV still photographs; three-dimensional maps were generated in Qimera 2.2.3 (https://qps.nl/downloads/qimera/), and two-dimensional maps were produced in ArcGIS Pro 3.5. No satellite imagery is included.

## Supplementary Information

Below is the link to the electronic supplementary material.

Supplementary Material 1

## Data Availability

The datasets used and/or analysed during the current study are available from the corresponding author on reasonable request.
