# Supplementary material for: Structural control and depth clustering of extensive hydrothermal venting on the shelf of Milos Island
Source: Sci Rep. 2025 Nov 27;15:42359. doi: 10.1038/s41598-025-26398-y (PMC12660848; doi:10.1038/s41598-025-26398-y)
Supplement: Supplementary file 1 — Supplementary Material 1 [file 41598_2025_26398_MOESM1_ESM.pdf]

Structural Control and Depth Clustering of Extensive Hydrothermal Venting on the Shelf of Milos Island

Paraskevi Nomikou[1*], Konstantina Bejelou[1], Andrea Koschinsky[2], Christian dos Santos Ferreira[3,4], Dimitrios Papanikolaou[1], Danai Lampridou[1], Stephanos P. Kilias[1], Eirini Anagnostou[2,3], Marcus Elvert[3,4], Clemens Röttgen[3], Joely M. Maak[4], Alissa Bach[3], Wolfgang Bach[3,4], Areti Belka[1], Evgenia Bazhenova[4], Karsten Haase[5], Charlotte Kleint[4], Effrosyni Varotsou[1], Palash Kumawat[3], Erika Kurahashi[2], Jianlin Liao[2], Eva-Maria Meckel[2], Ignacio Pedre[2], Wiebke Lehmann[5], Enno Schefuß[4], Michael Seidel[6], Sotiria Kothri [1] & Solveig I. Bühring[4]

[1] Department of Geology and Geoenvironment, National and Kapodistrian University of Athens, Athens, Greece

*corresponding author email: evinom@geol.uoa.gr

[2] School of Science, Physics & Earth Sciences, Constructor University Bremen

[3] Faculty of Geosciences, University of Bremen, Klagenfurter Str. 4, 28359 Bremen, Germany

[4] MARUM- Center for Marine Environmental Sciences, University of Bremen, Leobener Str. 8, 28359 Bremen, Germany

[5] GeoZentrum Nordbayern, Friedrich-Alexander-University Erlangen-Nuernberg, Erlangen, Germany

[6] Institute for Chemistry and Biology of the Marine Environment (ICBM), Carl Von Ossietzky University of Oldenburg, Oldenburg, Germany

This file contains:

the link for a supplementary video

(https://nc.uni-bremen.de/index.php/s/kBERaaFAzb8DbQz?dir=/&editing=false&openfile=true),

the link for a 3D model of the vent fields, hosted on the PANGAEA platform (https://doi.pangaea.de/10.1594/PANGAEA.974816)

and the supplementary Figures 1-5 as follows:

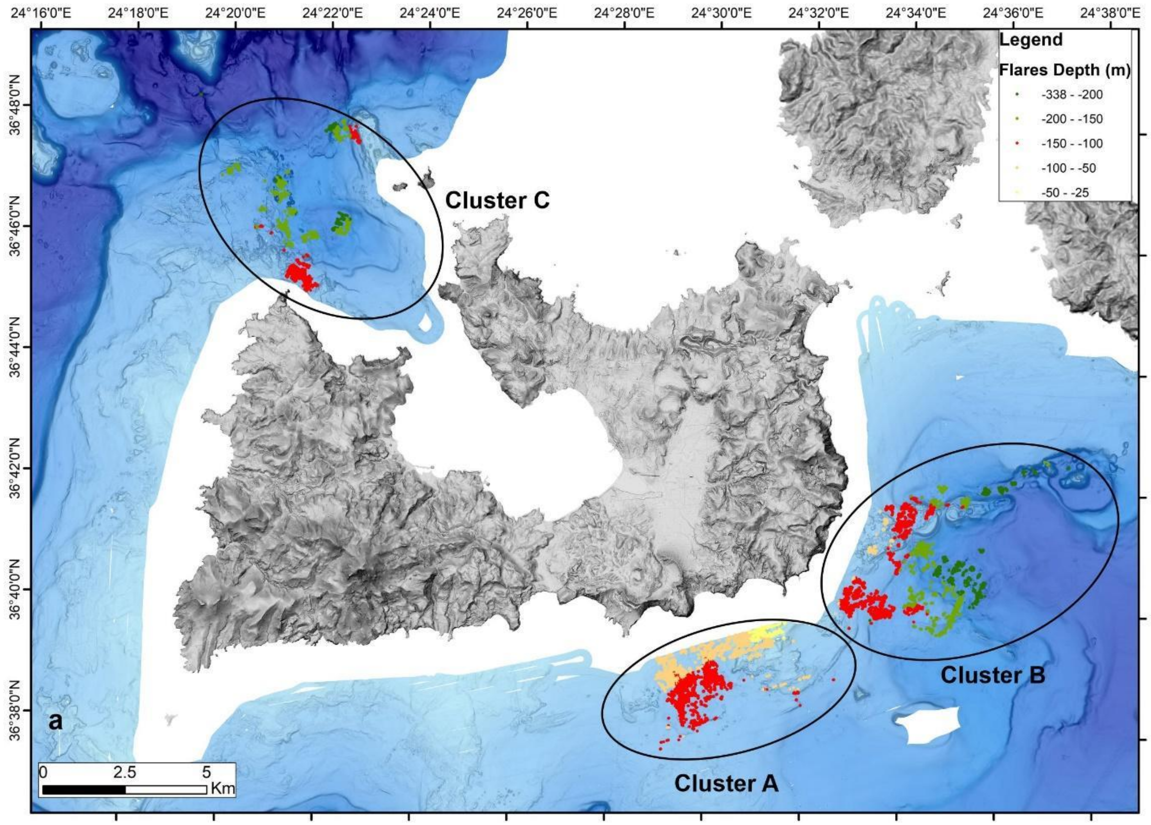

Supplementary Figure 1. Bathymetry map showing all the identified flares classified by depth.

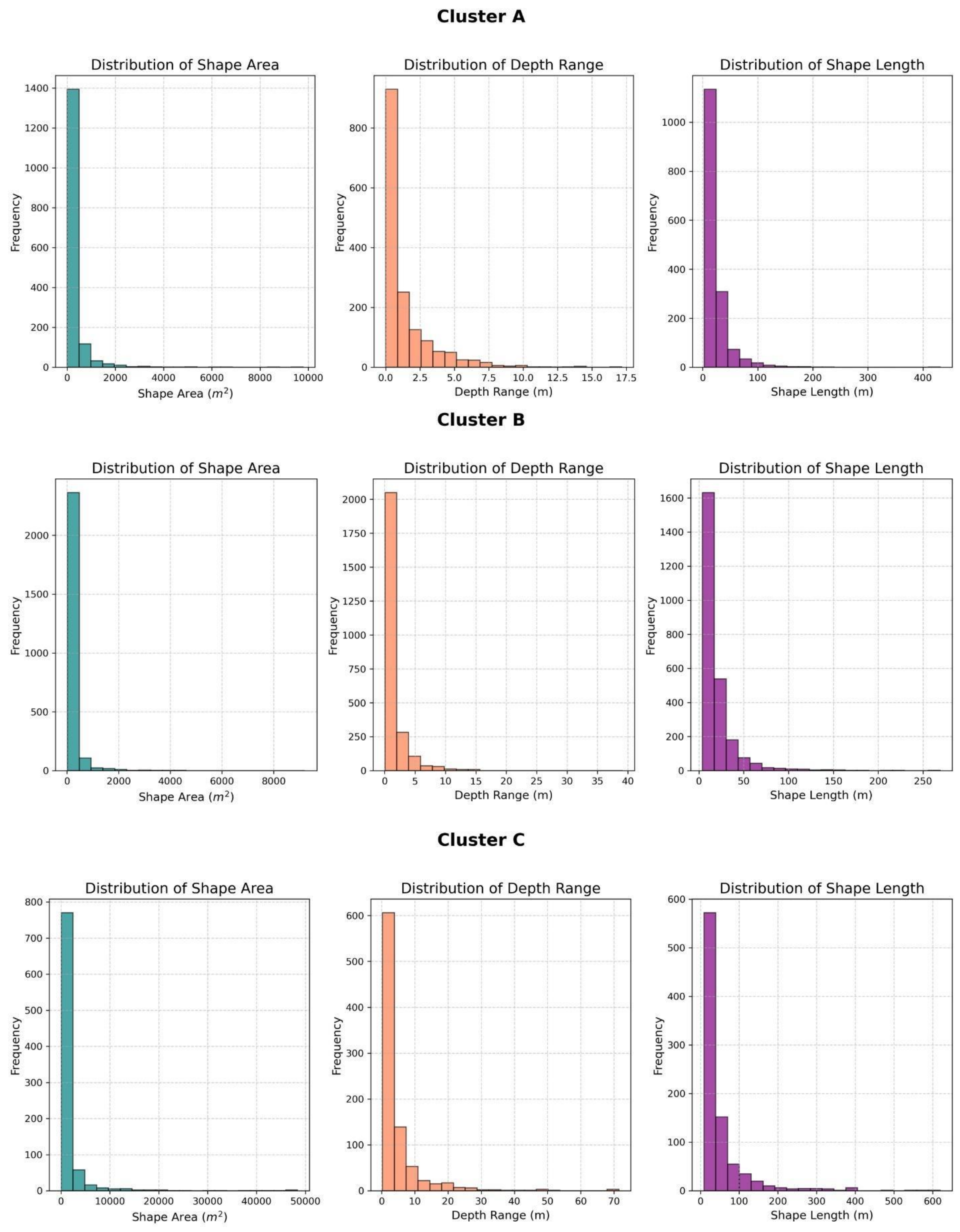

Supplementary Figure 2. Histograms showing the distribution of area (m^2), depth range (m) and length (m) of the depressions identified within all the clusters. A prominent feature identified across

the surveyed area is the presence of depressions, many of which appear to be correlated with fluid expulsions. These depressions have been observed within all clusters, varying significantly in size and depth. Two main types of depressions associated with gas release have been distinguished. The first type is located in the shallower areas along the coastline, often related to the rugged topography. The second type, which is found at greater depths, comprises isolated pockmarks. Notably, the most significant depressions are located in Cluster C, in the northwest part of Milos, where a depression spans nearly 400 meters in size and reaches a depth of 70 meters. In contrast, the depressions in Cluster A are significantly smaller, with one of the largest pockmarks measuring 100 meters in length and 7 meters in depth.

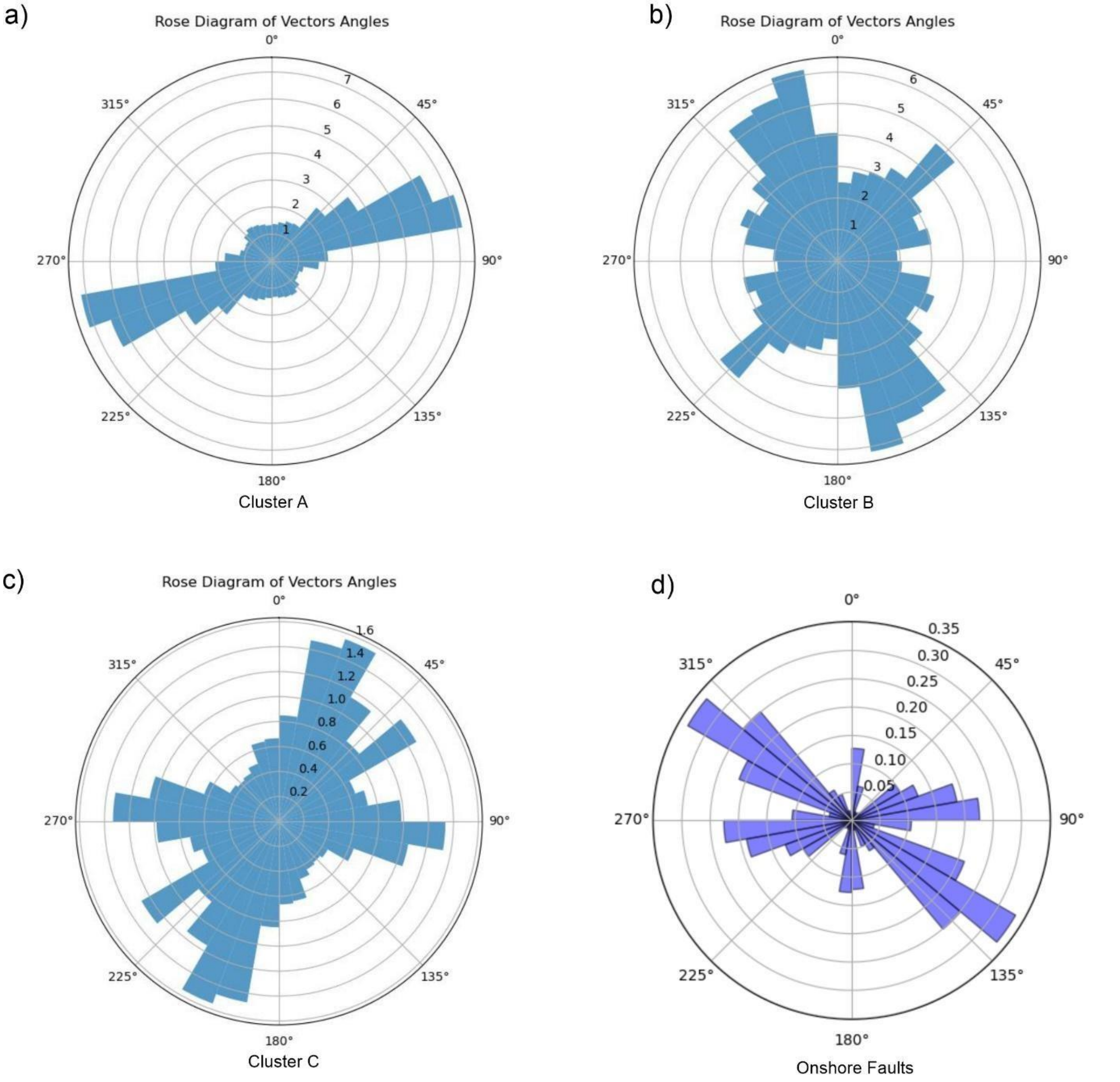

Supplementary Figure 3. a) Cluster A rose diagram of the alignment of the points. b) Cluster B rose diagram of the alignment of the points. c) Cluster C rose diagram of the alignment of the points. d) Onshore faults rose diagram. In addition to the geomorphological analysis, we also

conducted a Fry analysis to identify potential point patterns and explore any tectonic implications concerning the onshore geological regime. The spatial arrangement of gas flares within Cluster A exhibits a clear alignment with the coastline, primarily trending SW-NE, with a less pronounced N-S orientation. Two main orientations can be identified in Cluster B: the predominant NNW-SSE trend and a secondary NW-SE trend. The latter is closely associated with a linear morphological feature extending seawards, characterized by alternating crater-like depressions and vents. Cluster C presents a more complex pattern, with the major trend oriented nearly SW-NE and a secondary trend striking W-E. When comparing these rose diagrams with the onshore fault structures, there is a clear correlation between the fault orientations and the alignment of the gas fields. Specifically, all clusters have been influenced by faults trending SW-NE. Cluster B also shows a strong correlation with the three main fault zones traversing the island, which trend NW-SE. These fault zones are linked to major tectonic episodes that formed tectonic horsts and grabens associated with the NW-SE extensional stress field. Finally, the W-E trend identified in Cluster C can correlate with the nearly WSW-ENE trending faults, predominantly observed in the western part of the island.

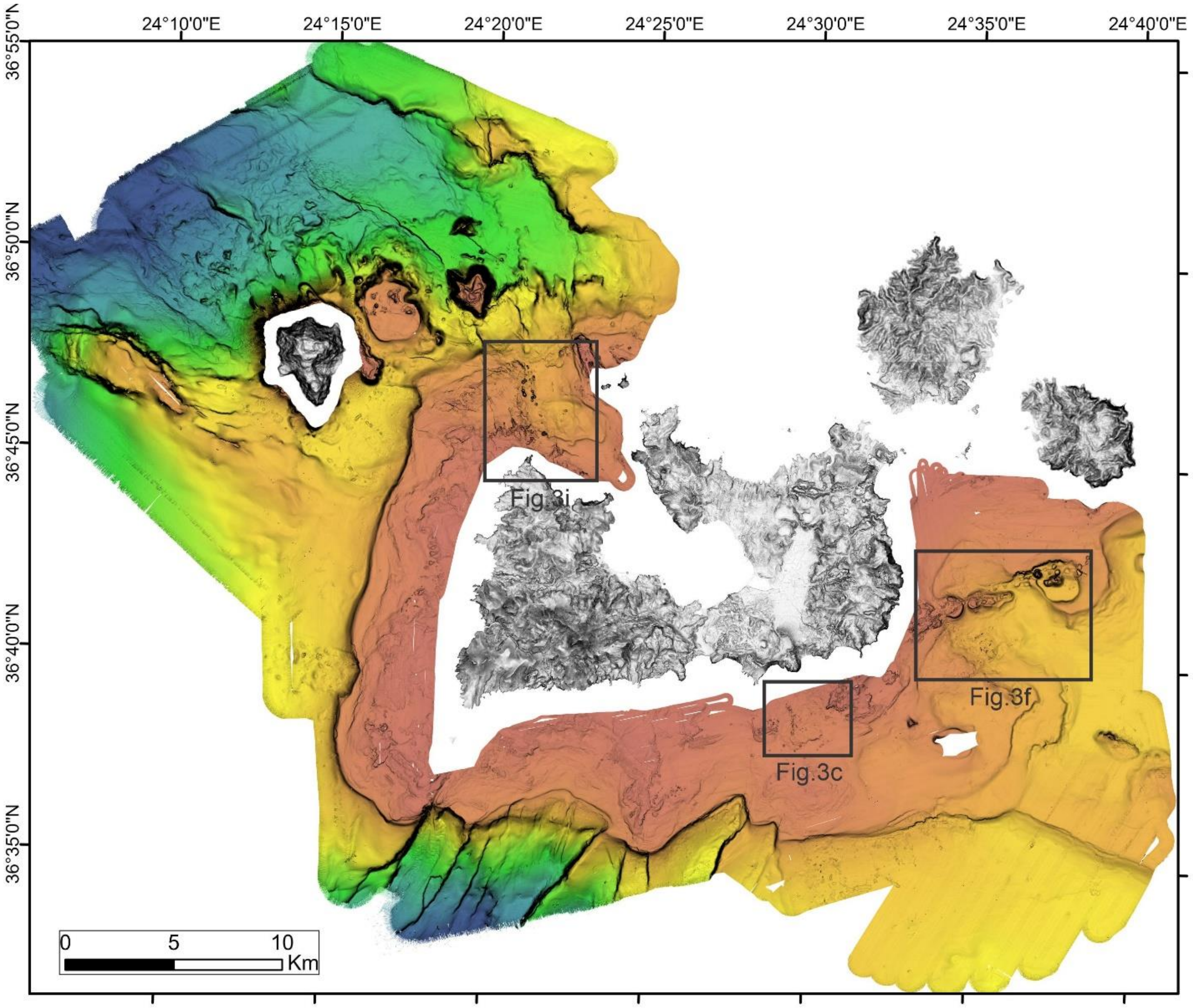

Supplementary Figure 4. Bathymetric map showing the locations of Figures 3c, 3f, 3i.

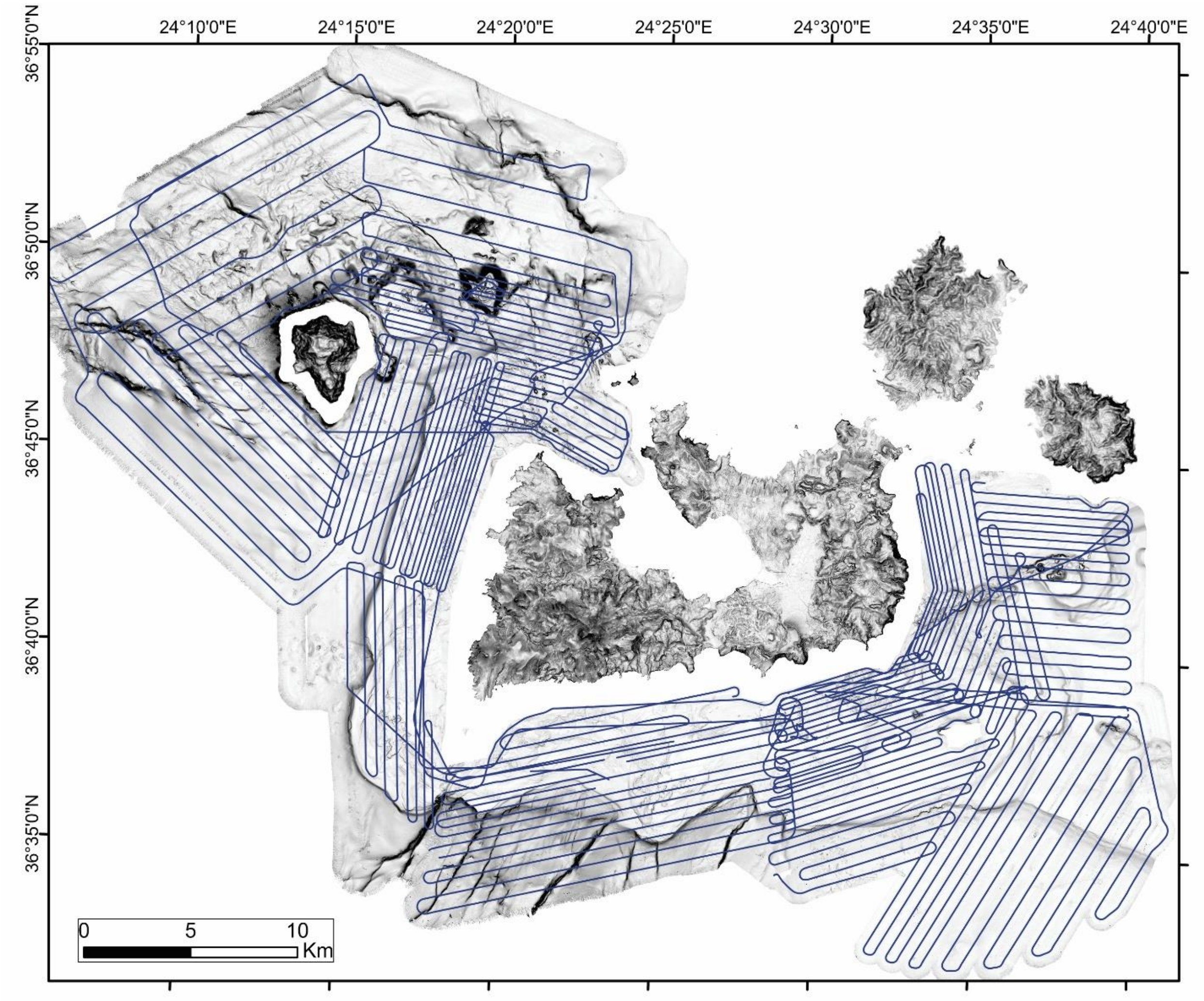

Supplementary Figure 5. Map showing all the acquired seismic data during the cruise.
